# Mindfulness-Based Interventions for In-Patients With Schizophrenia Spectrum Disorders—A Qualitative Approach

**DOI:** 10.3389/fpsyt.2020.00600

**Published:** 2020-06-26

**Authors:** Kerem Böge, Almira Karadza, Lukas M. Fuchs, Felicitas Ehlen, Thi Minh Tam Ta, Neil Thomas, Malek Bajbouj, Eric Hahn

**Affiliations:** ^1^ Department of Psychiatry and Psychotherapy, Charité - Universitätsmedizin Berlin, corporate member of Freie Universität Berlin, Humboldt-Universität zu Berlin, and Berlin Institute of Health, Berlin, Germany; ^2^ Clinical Psychology and Psychotherapy, Psychologische Hochschule Berlin, Berlin, Germany; ^3^ Institute of Sociology, Freie Universität Berlin, Berlin, Germany; ^4^ Department of Psychiatry, Jüdisches Krankenhaus, Berlin, Germany; ^5^ Centre for Mental Health, Swinburne University of Technology, Melbourne, VIC, Australia

**Keywords:** mindfulness, schizophrenia spectrum disorders, in-patient, qualitative research, positive symptoms, negative symptoms

## Abstract

In recent years, mindfulness-based interventions (MBI) have gained clinical relevance in the treatment of patients with schizophrenia spectrum disorders (SSDs). High symptom burden, long durations of hospitalization and high rehospitalization rates demonstrate the severity and cost-intensity of these disorders. MBIs have shown promising treatment outcomes in a small number of trials, primarily taking place in English-speaking countries. The current study aims to explore mechanisms and processes as well as adverse effects of MBIs on in-patients with SSDs in a German university hospital setting. A qualitative design based on inductive thematic analysis accompanied by quantitative assessments was chosen. A semi-structured interview guide was developed by psychiatrists and psychologists to assess patient experiences, perceptions, thoughts, and feelings during and after taking part in a MBI. Twenty-seven interviews were conducted between September 2017 and October 2018 with in-patients who are diagnosed with schizophrenia or schizoaffective disorder. Rater-based questionnaires, such as the Positive and Negative Syndrome Scale (PANSS), Montgomery Asberg Depression Rating Scale (MADRS), and Psychotic Symptom Rating Scales-Auditory Hallucination (PSYRATS-AH) were administered at baseline to collect clinical outcomes. Qualitative analysis revealed two domains: content and function. In the first domain related to *content* with the *core elements* “detachment and rumination”, “presence and getting lost”, “non-judgment and judgment”, and *effects* with “emotions”, “cognition”, and “symptom changes”. A second domain related to *function* was extracted, including the relevance of *perception of context* and *transfer to everyday life*. Overall, improvements concerning cognition, distress, and psychopathology were detected, while no adverse effects, such as increased psychotic symptoms, were revealed. As the first study of its kind, mechanisms, processes, and the safety of MBIs were explored and confirmed in a sample of German in-patients with SSDs. The results of this qualitative study are in line with recent findings on MBIs amongst patients with psychotic disorders from other countries. Results lay the ground for future research to focus on the systematic study of MBIs in large samples, its treatment processes, outcomes, and effectiveness for in-patients with SSDs.

## Introduction

In Germany, schizophrenia spectrum disorders (SSDs), including schizophrenia and schizoaffective disorder, account for up to 13% of patients in psychiatric hospitals (1). SSDs are defined by the presence of a broad range of symptoms (2), which often require expensive and lengthy in- and outpatient treatment. Symptoms representation is multi-faceted and can primarily be distinguished along with three main domains (3, 4): (1) positive symptoms, which are exemplified by delusions, hallucinations as well as disorganized thinking and behavior (2, 3); (2) negative symptoms, which are signified by a diminished emotional expression, anhedonia, blunted affect and scarcity of speech (4); and finally (3) there is profound evidence suggesting that SSDs can be characterized by a broad range of cognitive impairments (5, 6). Cognitive deficits are experienced through executive dysfunctions in processing speed, a decrease in attention control, and loss in semantic memory (7). Moreover, 50% of all affected individuals display comorbid symptoms of depression (8). It has been estimated that in 65% of individuals with SSDs, symptoms of anxiety are also present (9). Therefore, the estimated treatment costs of SSDs for the German public health system range between 9.63 and 13.52 billion Euros, indicating its cost-intensity (10).

International guidelines recommend evidence-based treatment options such as psychopharmacology, cognitive-behavioral therapy (CBT), and psychosocial support for the treatment of SSDs (11, 12). Several limitations of antipsychotic pharmacological treatments have been acknowledged (13), as meta-analyses estimated their effectiveness, primarily measured on positive symptoms, with overall medium effect sizes (14). The current state of research highlights the effects of pharmacological approaches mainly on positive- and depressive symptoms, although long-lasting improvements on negative symptoms, cognitive performance, social functioning, and overall quality of life remain unsatisfactory (14–17). Currently, it has been assumed that only 29% to 38% of patients achieve full symptomatic remission within one year (18–20), with rehospitalization rates of 40% within the first and second year (21–23). In Germany, the average treatment duration for in-patients with SSDs is between 21 and 32 days (24).

Furthermore, due to the existing structural, organizational, and financial barriers, the implementation of professional care, including psychotherapeutic and psychosocial interventions during hospitalization and aftercare, has been reported as insufficient (25, 26). Concerning organizational barriers, it has been noted that only about 1% of patients with SSDs received psychotherapy in Germany (27–29). On a structural level, national psychotherapy guidelines only covered accessory and residual symptoms, however, not direct symptoms of SSDs (25, 29). Moreover, insurance companies denied psychotherapy requests for SSDs more frequently in comparison to other mental disorders (25). In recent years, however, contextual changes in favor of psychotherapy for SDD have been introduced in Germany. The most recent evidence-based German national guidelines (S3-Leitlinien) (30) have been amended to now include psychotherapy as a central pillar in the treatment of SSDs, regardless of illness progression.

Moreover, through comprehensive efforts of multi-stakeholder treatment options could be partially increased (outpatient psychotherapy up to 3%) by facilitating state-of-the-art care, anti-stigma efforts, and training of evidence-based psychotherapy for SSD to overcome multiple barriers (29). Despite these changes, there still remains a huge gap in treatment options, therapy manuals, and well-trained psychotherapists in CBT for SSDs in both in- and outpatient settings (31, 32). For the in-patient settings, however, only a minority of patients receive individual CBT in addition to psychotropic medication due to structural and financial barriers, whereas group therapies, for instance, metacognitive training and psychoeducation, are more commonly applied (31, 32). The implementation of individual CBT remains difficult for SSDs due to prolonged treatment durations and high human capacity costs (26, 33). Lastly, CBT recommendations are more frequently based on trials conducted in outpatient settings (34–36), limiting their practicability and applicability to the direct in-patient treatment. Since patients with recurrent or chronic psychotic illnesses spend considerable time in the hospital and outpatient treatment for relapse-prevention, novel psychotherapeutic approaches are needed that achieve symptoms reduction and overall quality of life. Moreover, those interventions should improve efficiency in short- and long-term treatment by becoming more economically reasonable and easy to implement in routine care. Although cognitive-behavioral therapy (CBT), in combination with pharmacological treatment, is currently the evidence-based treatment of choice for patients with SSDs, third-wave cognitive-behavioral approaches are advancing in psychotherapy research and practice (37, 38), covering domains, such as mindfulness, acceptance, and cognitive diffusion (39).

The practice of mindfulness is deeply embedded in eastern meditation traditions. In Buddhist traditions, the term mindfulness originates in the Pali term *sati* and can be translated by bare attention, “keeping faithfully to its post of observation, watches calmly and without attachment the unceasing “(40), even though there have been extensive debates concerning its broader meaning and application (41). Essentially, the practice of mindfulness is understood as the presence of spirit and form - an essential part of Buddhist practice for purifying consciousness, sharpening wisdom, calmness, and constitutes the first factor of seven to achieve enlightenment. Mindfulness, as today's secular and therapeutic exercise in self-awareness has gained extreme popularity in the Western world in the past decades. Mainly, due to the work of Kabat-Zinn, who introduced mindfulness apart from Buddhist religious affiliations into clinical psychology and psychotherapy. Even though there have been ongoing debates about the concrete definition and concept of mindfulness, primarily, it has been defined as a state of clear consciousness with contentless attention (42). Furthermore, it involves a non-judgmental moment-by-moment awareness of thoughts, feelings, and sensations characterized by inner acceptance. By practicing mindfulness, one is empowered to increase the awareness of one's own stream of consciousness from a distant observing point, the meta observer (37). This practice consequently encourages a mental state depicted by qualities of nonjudgment, nonreactivity, detachment, acceptance, and compassion to oneself and among others. In psychotherapeutic approaches, it is therefore used as a technique to foster a mindful and accepting stance towards distressing symptoms and experiences instead of restructuring, changing, or eradicate these.

In recent years, MBIs have gained substantial scientific evidence across a range of disorders (43). They have been endorsed by national clinical guidelines, such as the UK National Institute for Health and Clinical Excellence (NICE) and the Canadian Network for Mood and Anxiety Treatments (CANMAT) for the treatment of affective disorders (44). Similarly, national guidelines in Germany emphasize MBIs for the treatment and relapse-prevention of patients with affective disorders (DGPPN, 2015). Currently, the evidence for mindfulness-based interventions (MBI) in the treatment of various psychiatric and somatic disorders is increasing yearly, including the treatment of other ailments such as chronic pain (45–47), anxiety (48, 49), depression (50, 51), and eating disorders (52, 53). Latest research has shown that even short-term MBIs ranging from one to four sessions can crucially reduce depressive symptoms and pain sensation (54, 55). Therefore, MBIs indicate transdiagnostic clinical utility by targeting individuals' relationship towards symptoms instead of their remission.

Although MBIs have been used for the treatment of a variety of mental disorders since the 1970s, research on MBIs in SSDs remains scarce (56, 57). One reason for this might be the concern that these interventions could be harmful and cause the exacerbation of psychotic symptoms (58). Various circumstances of adapted meditation practices, such as several hours of intensive meditation, sleep deprivation through meditation, or concentrative forms of meditation, have been reported to be potentially safe for patients with SSDs. However, these findings are based mainly on uncontrolled case studies (59–61). Consequently, research and clinical trials have deliberately focused on careful adaptations and changes in the implementation of MBIs for patients with SSDs. In recent years, a small number of randomized controlled trials have been conducted, showing that adapted MBI protocols can be successfully, safely, and effectively applied for the treatment of patients with SSDs. These adaptions comprised the shortening of mindfulness exercises, minimizing periods of silence, include basic anchoring exercises, smaller group sizes (< 6), as well as MBIs delivered by experienced mindfulness practitioners (62). Current meta-analyses demonstrated that those MBIs protocols are effective in reducing psychotic symptoms (57) as well as depressive symptoms in SSDs (57, 63), while results are mixed for negative symptoms (57, 64–67). Additional studies of MBIs have revealed an improvement in social functioning (68) and self-esteem (69). Furthermore, MBIs proved to reduce rehospitalization rates, up to one-fourth compared to participants that received treatment as usual (TAU) (68, 70–73). A study also reported shorter in-patient treatment durations from an average of 18.2 days with TAU to 11.8 days per year in the group with MBIs at an 18-month follow up (72). Furthermore, a study demonstrated that one year after a brief four-session MBI, effects remained to be clinically significant (74).

In general, recent research has shown that MBIs have been successfully applied in group settings and may require less specialized therapeutic skills or less prolonged professional training than using CBT in the treatment of patients with psychotic symptoms. Thus, an implementation is possible in shorter periods, while significant effectiveness has been shown within just 6–10 sessions, and patients have shown consistently high commitment (56, 57, 75, 76). This makes MBIs a cost-effective treatment option, also potentially suitable for the in-patient setting, in which frequently only a limited time frame for psychotherapeutic interventions is available, and group therapies are commonly implemented. In conclusion, the promising effects of adapted MBIs in SSDs have been confirmed in four meta-analyses comprising total psychotic symptoms, positive- as well as negative symptoms, depression, mindfulness, and rehospitalization rates and duration (56, 57, 77, 78). Moreover, two of these explicitly stated that MBIs in group format showed relatively larger effects compared to individual sessions (57, 78).

The two main mindfulness-based group format protocols which have been examined so far include the person-based cognitive therapy protocol designed for community settings in the UK, and the mindfulness-based psychoeducation protocol developed and evaluated in Taiwan, Hong Kong, and other parts of mainland China. Hence, there is a need to consider adopting their applications to other countries' health systems and cultures. To the best of the author's knowledge, no manual for German settings has been developed yet. For an adequate adaptation of MBIs for the treatment of SSDs, a careful assessment of the mechanisms and processes of a brief MBI for in-patients in the German language and health system is imperative. In Germany, the mental health system displays structural, organizational and personal differences in comparison with the UK, Hong Kong or China. While the UK has a strong emphasis on community-based approaches, in Germany, in-patients with SSD in open wards tend to be less acute in terms of positive symptoms. Moreover, they have substantially longer treatment duration (on average 37.6 to 40.6 days, in university hospitals up to two-months are possible) (79). Most wards are open, and patients stay voluntary. Therefore, German hospitals offer unique structural settings for psychological interventions such as MBIs which can successfully be implemented in group format (78). Concerning the treatment length in Germany MBIs enduring for approximately four weeks might be especially suitable, even though an in-patient trial from the UK showed effects after 1–3 sessions (80).

To this aim, a qualitative approach was selected to achieve an optimal understanding of the observed clinical outcomes in the study. Semi-structured interviews were chosen for their collaborative nature and their capacity to strengthen the connection between researchers and patients with SSDs (81). This approach also allows for gaining in-depth insights into the mechanisms and processes behind MBIs (82). In exploring the patients' thoughts and perceptions, qualitative research is crucial to understanding the mental health situation, specific service needs, and adaptions of MBIs. Therefore, the current study explores the responses of in-patients with SSDs to adapted MBIs (62) to understand possible mechanisms and processes in a German university hospital setting for the first time.

## Methods

### Design

A qualitative design was chosen with additional quantitative measures. Initially, rater-based measures were conducted prior to the group therapy (baseline) in order to assess a variety of clinical outcomes, including positive-, negative- and depressive symptoms of SSDs, as well as to examine exclusion criteria. Furthermore, these quantitative clinical outcomes enabled the comparability of the present sample characteristics in future trials and were further used to contextualize results according to the symptoms representation. However, *a priori* knowledge did not inform or alter the analytical process of qualitative data. The primary focus of the study was on the qualitative assessment of semi-structured interviews after each therapy session to explore experiences of psychiatric in-patients with SSDs during and after MBIs. Thematic inductive analysis (83), also defined as “a method for identifying, analyzing and reporting patterns (themes) within data” (84) was chosen for three main reasons: (1) The processual collecting of data and the intertwined analytical process, which begins during data collection and in itself informs the research process, allows a more focalized approach to issues of mental health (85). (2) Thematic analysis' well-structured step-wise process of finding, reviewing, and redefining themes is a transparent, hence reliable and valuable tool for inductive research in a team. The structured way in which inductively elicited themes are collated from repeating, and recurrent patterns in the data additionally allow for generalized conclusions regarding the effects of MBIs from the personal experiences and implications of our participants (84, 86). (3) At the same time, direct quotes and detailed descriptions obtained from the participants provide valuable and valid in-depth insights into the topic at hand (87, 88).

### Procedure

**Figure 1:**
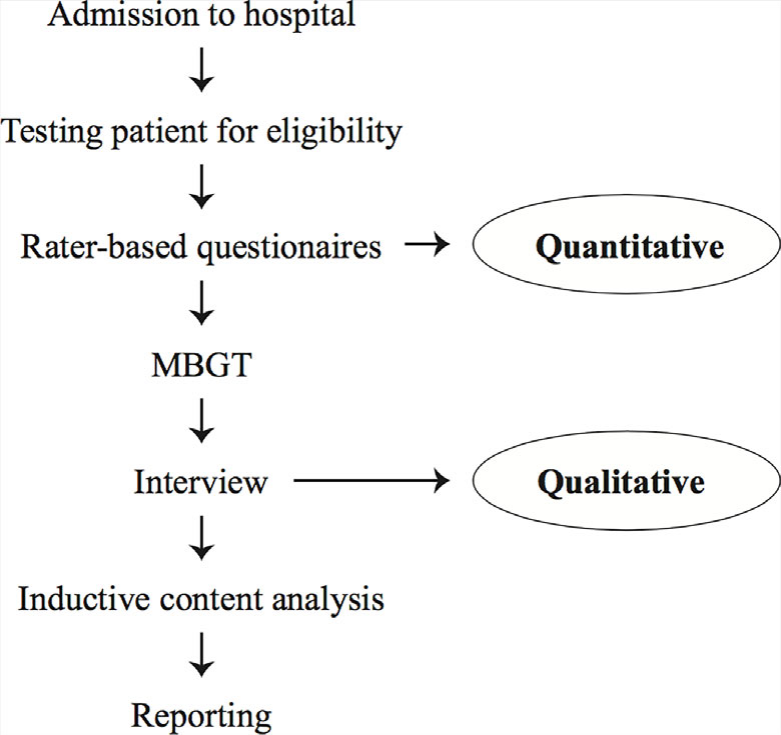
Study flow diagram.

Recruitment took place at the Charité – Universitätsmedizin Berlin, Department of Psychiatry and Psychotherapy, Campus Benjamin Franklin. Patients who underwent in-patient or day-care treatment were asked to participate. Participants fulfilling inclusion criteria, as shown in **Table 1**, were offered to attend the mindfulness-based group therapy (MBGT). Before the first session, multiple rater-based measures were given to each consenting participant to verify inclusion criteria and to obtain current psychopathology severity corresponding to the international diagnostic criteria based on the ICD-10. After study participants had attended one or two sessions, the semi-structured interviews were conducted to collect the qualitative data. **Figure 1** displays the flowchart of the present study. Participants were informed about the purpose of the study and written informed consent was obtained. The study was approved by the ethical committee of Charité – Universitätsmedizin Berlin, Germany.

**Table 1:** Inclusion and exclusion criteria.

Inclusion criteria
Diagnosed with a schizophrenia spectrum disorder by a qualified psychiatrist according to ICD-10 criteria Informed consent to participate in the study Aged between 18 and 65 years
Exclusion criteria
Severe psychotic symptoms (PANSS, P-Scale any single item > 5) Acute suicidality (MADRS, Item 10: Suicidality > 3) Acute substance use or substance abuse (except nicotine) according to ICD-10 criteria Severe traumatic cerebral injury in the past

P-Scale, Positive Syndrome Scale of Positive and Negative Syndrome Scale (PANSS).

### Quantitative Questionnaires

Psychotic symptoms were assessed through two dimensions of the Positive and Negative Syndrome Scale (PANSS) (89), 1) the Positive Syndrome Scale and 2) the Negative Syndrome Scale. The PANSS is a widely used and established rating scale to capture the presence and severity of psychotic symptoms within the 7 to 14 days before the assessment. Each scale contains seven items, which can be rated on a scale from 1 to 7, with higher values representing higher impairment. The Positive Syndrome Scale contains items about delusions, conceptual disorganization, hallucinations, excitement, grandiosity, suspiciousness, and hostility. The Negative Syndrome Scale assesses blunted affect, emotional withdrawal, poor rapport, passive withdrawal, difficulty in abstract thinking, lack of spontaneity, and stereotyped thinking. Scores are obtained by summing items for each sub-scale separately. In the norm sample of the PANSS, reported means were 18.2 for the Positive Symptom Scale and 20.0 for the Negative Syndrome Scale (89). Previous research demonstrated good reliability, predictive validity, and comparability with the Brief Psychiatric Rating Scale (90).

The Montgomery–Åsberg Depression Rating Scale (MADRS) (91) is a rater-based assessment for the severity of depressive symptoms in the seven days preceding the assessment. The scale has ten items that represent the main symptoms in depression and are sensitive to change. Excellent inter-rater reliabilities (0.89–0.97) and validity were also shown (91). Every item can be rated from 0 “no impairment”, to 6 “extreme impairment”. This results in a total score of 0 to 60, with higher scores representing the severity of the impairment: 0–6 absent symptoms, 7–19 mild depression, 20–34 moderate depression, and > 34 severe depression (92).

If participants were experiencing auditory hallucinations, the Auditory Hallucinations Rating Scale (AHRS), a subscale taken from the Psychotic Symptom Rating Scales (PSYRATS-AH; (93) was conducted. The AHRS is a structured interview assessing various aspects of acoustic hallucinations such as frequency, loudness, and length within the past seven days. Psychometric properties were given with excellent interrater reliability (0.74–1.00), adequate internal consistency (Kendall's tau-*b* = 0.63–0.76), and validity (*r* = 0.81 with PANSS hallucination item) (94). Eleven items are rated on five-point scales with individual rating options. In total, sum scores range between 0 to 55 with higher scores representing higher impairment.

### Intervention

The group was implemented in the patients' health care program as an add-on to treatment-as-usual (TAU) at the Charité – Universitätsmedizin Berlin, Campus Benjamin Franklin. At the hospital, TAU consists of an interdisciplinary approach for in-patients with SSDs, including psychopharmacological treatment, psychotherapy (individual therapy and eight group therapies), social work assistance, occupational therapy, and exercise therapy.

The protocol for mindfulness-based group therapy (MBGT) (95) was developed through an inductive iterative process. Various MBI protocols were extracted from the current literature, carefully adapted for the needs of the patient group, and combined with novel developed exercises. Based on the interviews, participant experiences were reviewed throughout the study process. Feedback was analyzed, and in the next step, the exercises were re-adapted throughout an iterative process until finally, the SENSE manual emerged (95). The manual describes a four-week therapy program with three sessions per week, which were in detail one main session (60 min) and two consolidation sessions (co-sessions; 30 min). During the development and through the feedback several adaptions have been conducted on the protocol including shortening of mindfulness exercises (< 15 min), minimizing periods of silence (< 15 s), basic anchoring exercises, smaller group sizes (< 6), as well as MBIs delivered by experienced mindfulness practitioners which are in line with recommendations of Shonin, Van Gordon, and Griffiths (62).

In the SENSE (mindfulnesS for schizophrENia SpEctrum disorders) manual, every main session begins with a brief 10-minute recap of the last week in the context of mindfulness. In a second step, there is 15-minute interactive psychoeducation on the main elements of mindfulness (presence in the here-and-now, non-judgment, and detachment). Third, 10–15 min of mindfulness exercises are practiced based on the weekly topic (breathing, detachment, senses in nature, and body awareness). Lastly, there are 10 min of sharing experiences, based on the inquiry model (96) as well as 5 min for sharing individual aims for the next week. Co-sessions repeated the contents and exercises of the main sessions.

### Interviews and Data Collection

A semi-structured interview guide was developed after a thorough examination and review of the current literature in the field. Interviewers were instructed to carefully consider the respondents' state, disclosed by the sample's characteristics as provided by the quantitative data. The interview questions were discussed within the research team, consisting of certified psychiatrists, clinical psychologists, and a sociologist, to assess their content and ensure the use of simple and precise wording. At the beginning of each interview, participants were asked to provide demographic data, such as age, gender, housing situation, occupation, and first exacerbation of the disorder. A detailed description of the study process, the interview schedule, and questions are illustrated in **Table 2**.

**Table 2:** Study and interview schedule.

Determination of interview ability	5 min
Clarification of open question	5 min
Informed Consent	5 min
Rater-based measures	30 min
MBGT	60 min
Demographics	5 min
Semi-structured interview	20–50 min
*1. Do you have any previous experience with this exercise, or does it remind you of other exercises?*	
*2. What did you notice inside you during the exercise?*	
*3. How would you characterize this exercise?*	
*4. How and what did you perceive during this exercise?*	
*5. What prevented you from doing the exercise, or were there any difficulties?*	
*6. What did you manage well?*	
*7. Did the exercise affect you anyhow? If so, how?*	
*8. What can you take away for yourself from this exercise, and/or what did you miss?*	
*9. What did you perceive regarding your symptoms during and after the exercise?*	
Time for remarks and comments.	10 min

The data was collected between September 2017 and October 2018. Each interview consisted of nine open-ended questions and lasted between 25 and 45 min in total. The first interviews were conducted by KB, the principal investigator, who then trained two researchers (AK and FE) to conduct further interviews; multiple training sessions were held to ensure consistency. The training included discussion of the content, clarification of the open-ended questions, as well as pilot interviews. The interviews were conducted directly after the group therapy sessions. After the collection of socio-demographic variables and information on the life situation, participants were openly asked about their previous knowledge concerning MBIs as well as their experiences, perceptions, feelings, thoughts, and symptom perception before, during, and after the MBIs. The questions gave room for free elaborations about prior experiences and general knowledge about mindfulness. After encouraging participants to talk about their experiences, they were asked about their perceptions during and after the exercise. Questions included, for example, “How and/or what did you perceive during the exercise?” or “What did you perceive in relation to your mental health during/after the exercise? “to allow the participants to express their perceptions during the exercise freely, as well as to explore whether or not there was an exacerbation of psychotic symptoms. Each participant was interviewed once, at the latest, after the second session attended. To ensure data safety, anonymous transcripts were created, and the original interview documents were kept in locked filing cabinets, to which only the direct research team had access.

### Data Analysis

Following qualitative inductive analysis (86), data was thematically explored, analyzed, and compared step-wise to identify themes from the data (83) systematically. First, word-by-word coding (97), as well as open line-by-line coding (98), was used to reconstruct the data into keywords and contents and to compare them until concepts emerged, which allowed for categorization of the data. This fostered an iterative deepened understanding of the data in a constant critical and analytical process. In the second step, codes were thematically categorized into meaningful clusters (84, 99, 100). Finally, these groups were structurally and conceptually merged, facilitating an understanding of underlying domains (84, 97). The first two steps were conducted separately by two trained psychologists and researchers (AK and EE) using MAXQDA, a software program designed for computer-assisted qualitative data analysis. The joint final step integrated the separately found categories by discussing and relating them. During this stage, an independent qualitative researcher and sociologist (LF) joined the analysis process to review the procedure as well as the categorization of the analysis. In a final step, one psychologist (KB) and psychiatrist (EH) collaborated to review both content and formal categorization.

Concerning the consolidated criteria for reporting qualitative research (COREQ) guidelines, the authors acknowledge their unique background and perspectives to the analyses and aimed to bring these into consideration (101). The first author (KB) is a male psychologist, experienced mindfulness trainer (10 years) and a mindfulness and psychotherapy researcher. The second author (AK) is a female psychologist, beginning mindfulness trainer and mindfulness and psychotherapy researcher. The third author (FE) is a female senior psychiatrist, beginning mindfulness trainer, psychotherapy researcher. The fourth author (LF) is a male sociologist and researcher with a strong qualitative background focusing on mental health advocacy. The fifth author (TT) is a female senior psychiatrist and psychotherapy researcher with a strong focus on qualitative and quantitative transcultural psychotherapy. The sixth author (NT) is a male psychologist and psychotherapy researcher using qualitative and quantitative approaches. The seventh author (MB) is a male senior psychiatrist and psychotherapy researcher. The last author (EH) is a male senior psychiatrist and psychotherapy researcher. All discussions aimed to assess any possible influences of these backgrounds on the analysis process.

## Results

### Quantitative Outcomes

For the current study, 27 participants (22.2% women; 6 women and 21 men) with a mean age of 35.8 years (*SD* = 10.9) were included. Of these, 21 participants were diagnosed with paranoid schizophrenia and six with schizoaffective disorder. Nineteen participants were recruited from the ward for psychotic disorders, while eight participants were from the day-care ward. All participants were receiving psychotropic medication, which was not changed relevantly throughout the study process. Psychopathology assessed by PANSS, MADRS, and PSYRATS-AH showed that the participants, despite the exclusion of a severe psychotic episode, had a marked variety of symptoms at the beginning of the MBIs. On average, participants showed mild to moderate positive symptoms and moderate negative and mild depressive symptoms (89). Seven respondents reported experiencing acoustic hallucinations at the time of the study. A detailed summary of sample and symptom characteristics is shown in **Table 3**.

**Table 3:** Characteristics and symptom severity of the participants (n = 27).

	M	SD	Range
Age (years)	35.8	10.9	19–59
Illness duration (years)	10.6	8.9	1–40
Number of episodes	3.5	2.4	1–10
Number of hospitalizations	3.9	2.4	1–10
Length of last hospital stay (days)	78.8	32.1	21–138
PANSS, Positive Syndrome Scale	14.4	5.5	7–24
PANSS, Negative Syndrome Scale	20.4	7.3	7–35
MADRS	14.8	9.1	1–37
AHRS*	21.1	11.7	2–39

M, Mean; SD, Standard deviation, *n = 7 participants who reported hearing voices.

### Qualitative Results

**Figure 2:**
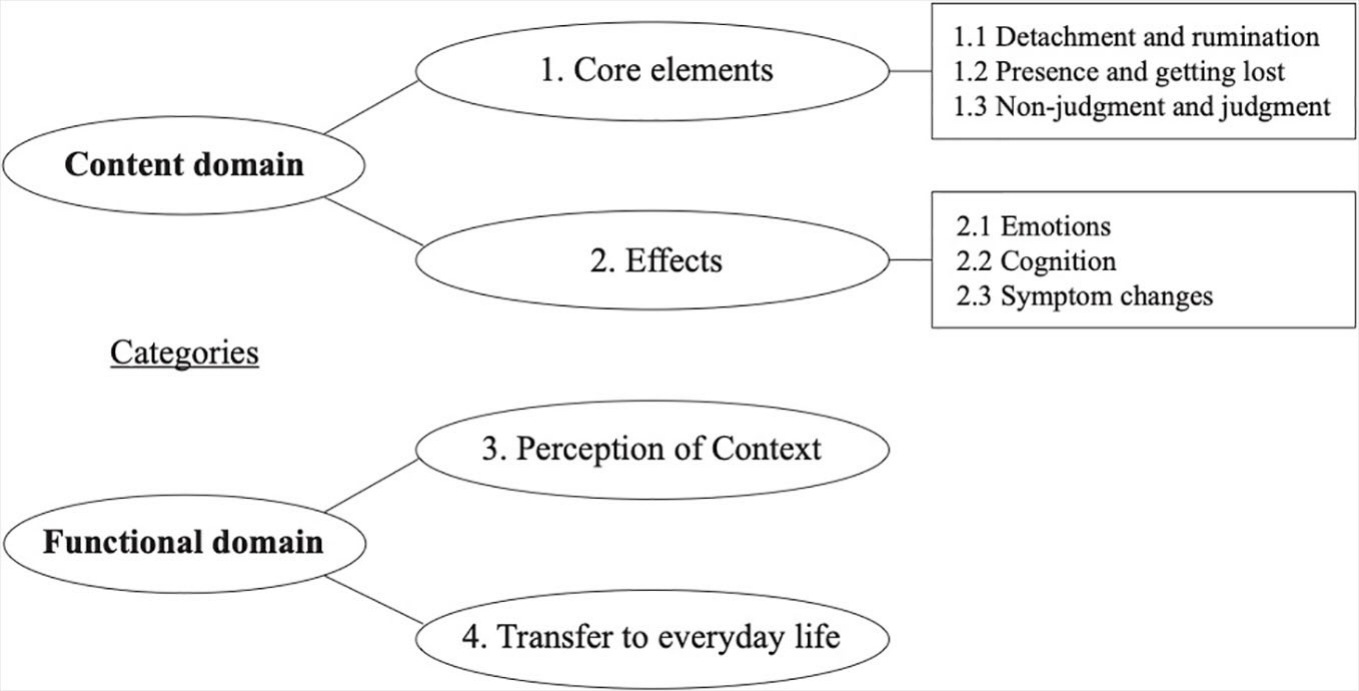
Summary of categories.

The 27 interviews were conducted and spread out over the following four sessions: “Mindfulness of breathing” (7), “Mindfulness of detachment” (7), “Mindfulness of senses in the context of nature” (6), and “Mindfulness of body awareness” (7). The thematic analysis of the interview data reduced the data step-wise, collated initial codes into themes, which were then redefined in relation to each other. All steps were conducted not merely by one researcher but by two or more. Based on the participants' experiences during and after the sessions, finally, two content and two functional subthemes were identified throughout the data analysis process. For the content subthemes, (1) *core elements* and (2) *effects* were generated, referring to essential transdiagnostic elements of mindfulness. For the functional subthemes, (3) *perception of context* and (4) *transfer to everyday life* described the implementation of the MBGT and participants' transfer of mindfulness associations to their daily life. Overall, the content domain, therefore, includes direct content matter and topics (*core elements*) about mindfulness as well as the *effects* on an individual subjective level. The functional domain, however, covers applicatory aspects such as the *perception of the context,* including external stimuli (therapist's voice, sound bowl, setting). Moreover, this domain described the practicability to utilize mindfulness in their everyday life (*transfer to everyday life*). A summary and brief description of the categories can be found in **Table 4** and **Figure 2**.

**Table 4:** Description of categories.

Content domain	
** 1. Core elements**	
1.1 Detachment and rumination	Attachment to emotions, thoughts, sensations
1.2 Presence and getting lost	Presence in the moment, past or future
1.3 Non-judgment and judgment*	Judgement about emotions, thoughts, sensations
** 2. Effects**	
2.1 Emotions	Emerged emotions
2.2 Cognition	Increased cognition and metacognitive effects
2.3 Symptom changes	Changes in symptoms awareness
** Functional domain**	
** 3. Perception of Context**	Awareness of sensory stimulation; sensing and experiencing contextual factors (Time, surrounding sounds, therapists, other group members, duration)
** 4. Transfer to everyday life**	Utility and practicability in daily life

*Latent category.

#### Content Domain

The content of both subthemes (1) *core elements* (2) and *effects* illustrate participants' experience of and through mindfulness. All three *core elements* were found to be coexisting mechanisms of mindfulness, whereas the *effects* were described as consequences of and in relation to the *core elements*.

##### Core Elements

Participants' responses were found to relate to three *core elements* of mindfulness: (1.1) *detachment*, (1.2) *presence,* and (1.3) *non-judgment*. While *detachment* and *presence* were usually named directly as mechanisms by the interviewees, the latent category *non-judgment* was mostly conveyed indirectly through expressed appreciations. Furthermore, the categories are found to be dimensional, with a wide range of responses being antagonistic to each other in participant's perceptions. The dimensionality of the categories results from the graduations of the participant's answers, respectively.

###### Detachment and Rumination

Concerning detachment, it was found that some participants were able to notice the appearance and disappearance of their thoughts, sensations as well as emotions:

“During the exercise, I noticed again and again that different thoughts came and then went away again. (Participant 12; Session: Detachment)

When the stress came up, many thoughts, such as tide and then slowly the tension, I could put off everything by the deep inhale and exhale. (Participant 09; Session: Breathing)”

In some instances, participants became aware of internal processes and were able to differentiate between passive observations, while others realized a sense of active engagement with inner processes. While in the first quote, emotions and thoughts are described passively, from a meta-observer position (“Came and then go…”), in the second quote, the participant assigned himself a certain level of agency (“I could …”).

However, some participants described further processes and displayed a feeling of identification between themselves and their psychopathology and the process of de-identification and de-centering:

“So, to realize that they [the symptoms] are only part of more in me. That I am not always just my symptoms that I can let go. (…) I am much more besides my symptoms. (Participant 10; Session: Breathing)”

These findings point towards participants' greater self-insight and self-understanding by detecting differentiated degrees of association between their psychopathology and self-concepts, such as self-esteem and recognition of oneself apart from symptomology. On the other side of the detachment dimension, participants showed patterns of rumination illustrated by a narrowed focus on impressions, thoughts, and emotions. It has been reported that perceived distress is a common therapeutic problem that prevents participants from opening themselves to further sensations, such as a change of perspective (for further discussion of this problem, see 102).

“I thought of the past and everything I have already experienced and been through. (Participant 15, Session: Body)”

###### Presence and Getting Lost

Participants referred to “Presence” as staying in the here and now, being in the present moment, and perceiving momentary sensations without actively changing anything. This can include thoughts, feelings, body sensations, as well as symptoms. Furthermore, participants described perceiving through their senses, such as feeling their breath or hearing noises in nature as a way of being present in the moment which resulted in changes of perception:

“Sometimes I wonder if everything has gone so well in the past. Then it helps to sense a feeling, to notice, to be fresh, to be new, and to be in the here and now. (Participant 17; Session: Detachment)

As I watched my breathing, I automatically changed my breathing. At first, I thought that was funny, but later I managed not to change it anymore, but only to perceive it. I felt better (Participant 07; Session: Nature)”

As shown in the first of the two quotes above, participants described not only pleasant perceptions, but also the awareness of negative thoughts and worries, unpleasant body sensations, emotions, or symptoms. While in those examples, participants were still able to draw attention to the present moment by focusing on the breath or another present sensation, others described how they lost the awareness of the present moment:

“I already knew before that the thoughts would digress. (Participant 10; Session: Breathing)”

“I found it hard to concentrate and started to muse. (Participant 11; Session: Detachment)”

Through the description of concentration problems, participants' difficulties in keeping awareness at the present moment were particularly evident. In the sense of a self-fulfilling prophecy, the participants got lost in ruminations about their perceived lack in concentration, and thus inhibiting their contact to the present moment:

“I felt time pressure because I did not know how long I could maintain concentration [ … ]. (Participant 04, Session: Nature)”

###### Non-Judgement and Judgment

This core element is characterized by the extent of judgment towards perceptions, thoughts, feelings, and other sensations. Here, some participants describe their impressions value-free, thus non-judgmentally, and without any categorization:

“I perceive my environment and my surroundings more and differently. I let them affect me more, and I do not imagine something directly. I try not to judge, but to perceive. I feel a little more. (Participant 10; Session: Breathing)”

Often, this category was presented only indirectly, through the description of negative impressions. The participants reported the appearance of unpleasant sensations, thoughts, and emotions, but at the same time invalidating the power of these negative impressions. This indicates that participants did not judge these negative impressions or had less judgment about them:

“Malaise was noticeable during the exercise but was not so unpleasant. (Participant 18; Session: Body)”

#### Effects

The category of effects is subdivided into references to (2.1) *emotions*, (2.2) *cognition,* and (2.3) *symptom changes*. For example, participants were asked how they perceived the exercise and which difficulties they had but also about symptoms they might have perceived during and after the session. It is noticeable that none of the participants described negative or adverse effects through the exercise, while improvements were described frequently.

##### Emotions

Although it was repeatedly stated during the theoretical conceptualization that mindfulness does not solely pursue the goal of relaxation or other positive sensations, participants nevertheless described those positive effects through their exercises. Asked openly about their perceptions during the exercises, participants described associations, such as the feelings of inner peace, relaxation, aliveness, or feeling happiness:

“Joy, peace, freedom, inner peace, relaxation. (Participant 02; Session: Nature)

It has moved something that cannot be put into words. Most likely ‘inner warmth', ‘authenticity', ‘being with you without having to do anything about it.' (Participant 07; Session: Nature)”

##### Cognition

Cognitive effects were described through the reported awareness of cognitive functioning, self-conceptions, and metacognition. As mentioned above, participants reported having concentration difficulties in their daily life. Thus, it was also reported in the interviews. However, no one described a negative effect on their concentration or other cognitive functions as a result of the exercises. Instead, not only an increase in the ability to concentrate is cited as the most frequent cognitive improvement (13 of 27 participants reported an increase), but also stimulation of imagination and creativity, as well as an increase in self-confidence, were mentioned.

Furthermore, metacognitive insights about their self-concepts and their functioning were reported:

“To have more concentration, because I could follow the tasks. In the process, I realized that I seem to be more involved with this than with other [therapy groups]. (Participant 25; Session: Body)”

“To concentrate completely on me. That went really well and has promoted my self-confidence. Then, I felt much more with myself. (Participant 09; Session: Breathing)”

##### Symptom Changes

At the end of every interview, participants were asked to describe their symptoms before, during, and after the exercise. While some described an improvement in symptoms, others reported no observable and subjective changes, however, what is noteworthy is that no one reported exacerbation or deterioration of symptoms during or immediately after the exercises.

“Before that, I was very tense and tight. Now I feel lightness, looseness, and internally flexible. (Participant 17; Session: Detachment)”

“I feel less stressed. I have felt less anxiety. It's okay how I am currently. (Participants 19; Session: Body)”

“Everything stayed the same. I do not notice any difference. The symptoms are actually always the same. I just learn more about how to live with them. (Participant 11; Session: Detachment)”

### Functional Domain

The two functional subthemes (3) *perception of context* and (4) *transferability to everyday life* refer to the general conditions and contextual factors as well as participants' direct experience, and awareness of sensory stimulation emerged through the intervention. These contain the applicability as well as helpful or disturbing circumstances for the participants. Frequently, participants commented on the intervention by emphasizing the perception of the context of the specific exercises and their usability in daily life.

#### Perception of Context

This category emerged through contextual variables, such as the therapist's voice, the instructions, or the use of a sound bowl as an anchor stimulus to direct distracted attention back to the perception in the here and now, which have been frequently named by participants. Moreover, the duration of the exercise or the influence and resonance with other group members have been mentioned. Therewith, participants stated for these factors an increased awareness of sensory stimulation, which positively influenced and supported therapeutic processes and mechanisms described in the previous categories of *core elements* and *effects*.

“Most of all, I was able to hear the therapist's voice. It encouraged me to pay more attention to myself and my inner life. (Participant 26; Session: Breathing)”

“But listening to the therapist's voice helped me. It is then as an anchor, and you try to focus on it. (Participant 11; Session: Detachment)”

“The gong was pleasant, relaxing. (Participant 23; Session: Detachment)”

In minor cases, persistent difficulty in carrying out the exercise was reported (7 out of 27 participants). For the most part, these were individual difficulties, such as concentration problems, exhaustion, or hunger. However, sometimes participants associated difficulties sensing and experiencing contextual factors, for example, due to disturbing, external perceptions such as noises from outside, the time of the day when the session is carried out, or the way exercises were instructed.

“Standing meditation exercise, which was exhausting. I prefer to sit or lie down. (Participant 22; Session: Nature)”

##### Transfer to Everyday Life

As the last category, participants reported the desire to transfer the learned mindfulness associations into the individual everyday life, demonstrating a vital psychotherapeutic success in the context of perceived self-efficacy. At the same time, participants expressed their ideas and visions on how to transfer, utilize, or form the practice of mindfulness and its application into their everyday lives.

“You can do this exercise regularly, and it can help you to cope better with the voices in your head. That's how you can detach from the voices. (Participant 11; Session: Detachment)”

“This exercise was much easier to implement [than Progressive Muscle Relaxation], and that's why I find it much better. I can do it at home alone. (Participant 13; Session: Breathing)”

“I was happy to do it. That was a good exercise for anxiety. I would like to take this as a technique and use it at work. (Participant 19; Session: Body)”

All participants reported having already heard of mindfulness in some form, or they named related associations from their everyday lives, even though none of the participants had any previous experience with MBIs:

“A reminder of how I have always sought and found tranquility and protection while walking through fields and meadows during my psychosis. (Participant 3; Session: Nature)”

## Discussion

The main objective of this study was to examine the experiences of in-patients with SSDs who took part in a brief MBI at a German university hospital setting. According to the authors' best knowledge, this is the first account of such a study in Germany. Collected data across all four different mindfulness modules were analyzed. Responses generated to a total of four subthemes within two domains, *content* and *functionality,* from which the effects of the MBIs can be understood. Whereas the *content domain* includes references to *core elements* and *effects,* each with three subcategories within the *functional domain*, participant awareness of possible *transfer to everyday life,* and the *perception of context* of the intervention is portrayed.

### Core Elements of Mindfulness

The first content domain *core elements* has three subcategories, including *detachment*, *presence,* and *non-judgment,* which overlap with key elements of mindfulness practice (42, 45, 103). However, participants' responses suggest that these core elements should be understood as dimensional categories, each with two poles along a continuum—*detachment and rumination, presence and getting lost,* or *non-judgment and judgment.* These polarities are reflected in Chadwick's (102) initially postulated framework model for patients with SSDs, which distinguishes between a mindful and distressing reaction to unpleasant psychotic sensations including voices, paranoid thoughts, or images. Along these poles, participants, on the one hand, seem to show a distressing reaction displayed by experimental avoidance leading to judgments. Therefore, increased rumination and worry occur taking participants back again to avoidant behavior—reflecting a vicious cycle restraint from action and getting lost in reaction.

On the other hand, participants learn a mindful response by allowing unpleasant psychotic experiences to come into awareness. This is depicted by actively turning towards the difficult sensations and cultivating acceptance for its current representation. With practice, individuals learn the process of letting go and develop more awareness (102). For the current study, as shown in subcategory 1.1 to 1.3 (*Detachment and Rumination, Presence and Getting Lost, and Non-Judgement and Judgment*), participants' responses seem to verify this process model and its dimensional categories since the type of reaction depend on the patients' degree of non-judgmental and detached awareness on symptom expression in the present moment. These findings are also consistent with existing descriptions of the main mechanisms within mindfulness practice in other clinical groups (96, 103), besides SSDs (102). For example, for patients with a Major Depressive Episode (MDE), Mindfulness-based Cognitive Therapy (MBCT) is a well-researched, established, and highly effective psychotherapeutic approach. Its underlying theory states that continuous deterioration of symptoms is experienced through the repeated associations between dysfunctional mood and patterns of negative and self-devaluating cognitive processes (104). In MDE the main aim and core skill that MBCT provides is the ability to inhibit potential relapses by first noticing and in a second step disengaging and detaching from mental states, which are characterized by self-perpetuating patterns of rumination, negative thoughts, and the tendency to identify with specific cognitions (105–107).

Moreover, similar treatment mechanisms and process-variables seem to play a role when implementing MBIs for patients with SSDs. Participants in the current study highlighted altered perceptions of the appearance and disappearance of their thoughts, sensations, and emotions described in subcategory 1.1 (*Detachment and Rumination*). Furthermore, results seem to elucidate the effect of MBIs on a behavioral and cognitive level since the exposure to adverse internal experiences leads to desensitization and, consequently, symptom decline (45). These subjective experiences include psychotic experiences, such as acoustic hallucinations, paranoid thoughts, or images, which lose their distressing and unpleasant sensation over time when mindfulness is cultivated by getting in touch with the presence and perceiving momentary sensations without active engagement. Overall, these results highlight mindfulness' transdiagnostic effects as similar, theoretically defined mechanisms seem to be crucial within the treatment process.

### Effects of Mindfulness

Three interconnected factors have been extracted from the interviews demonstrating the effects of mindfulness on *emotion, cognition*, and *symptom changes.* First, participants stated various effects on their emotional state during and after the MBI. These included states of deep relaxation, inner peace, euphoria, and happiness. Since ancient Buddhist times, mindfulness practice has been characterized by these conditions and is further associated with contentment and inner equilibrium. Present outcomes are in line with previous research, (65) with patients having SSDs revealing decreased negative symptoms and increased positive emotions as well as improved overall recovery. The main mechanism behind this effect may lie in the heightened levels of acceptance for psychotic experience and oneself. In another study, it was shown that mindfulness of positive symptoms was acting as a mediator between self-compassion and positive symptom severity and was negatively correlated with psychotic distress (108). Other studies demonstrated a clinical moderating effect of mindfulness for psychosis outcomes and self-compassion, indicating a synergistic relationship between the constructs (56). Similar associations between these factors have been shown in MDE, hinting at a transdiagnostic understanding of mindfulness (109). Moreover, participants described heightened feelings of authenticity, benevolence, and empathy towards themselves as well as to others as displayed in subcategory 2.1 (*Emotions*). These findings show that there is a distinct connection between mindfulness, compassion, and symptom outcome, even though the direction and interrelation between these factors have yet to be adequately studied. Future studies might examine this relationship more closely as both constructs, mindfulness, and compassion, possibly act as mediators regarding *emotions* and mindfulness.

As a second factor, participants named effects on *cognition,* which are exhibited by increased cognition, self-awareness, and empowerment through the practice of mindfulness. Some patients displayed increased levels of cognitive processes, such as concentration, as well as metacognitive insights into their self-concepts, as illustrated in subcategory 2.2 (*Cognition*). Among the first to address this, Tabak and Granholm (110) found supporting evidence concerning mindfulness effects on cognition as well. In their study, the authors revealed that in MBI, participants with psychosis showed significant improvements in processing speed and working memory, as well as increased attention/vigilance, verbal- and visual learning, which are most frequently impaired in patients with SSDs (111). Impaired cognition constitutes a major burden for patients with SSDs, and current treatment choices are insufficient. Larger controlled trials with extended mindfulness practices, similar to MBCT in MDE, may, in the future, broaden our currently limited knowledge regarding mindfulness and its effects on cognitive processes in SSDs. Besides improved awareness of cognitive functioning, participants in the present study highlighted increased self-conceptions and self-confidence after MBIs. Research amongst patients with MDE has shown that low metacognition is defined by an incapability to distinguish the self from negative thoughts, emotions, and sensations (96). Hence, the self is occupied with negative mental phenomena inhibiting the perception of a wider context of self. Relating and responding mindfully to symptoms allows for a detachment from these negative mental phenomena, alleviating distressing experiences and thus enabling more imagination, self-acceptance, and creativity in patients with SSDs as depicted in subcategory 2.2 (*Cognition*).

As a last factor illustrating the effects of MBI, participants described frequent *symptom changes* during and after sessions. In the present study, participants stated improvements on anxiety and depression levels, positive- and negative symptoms, as well as overall well-being as depicted in subcategory 2.3 (*Symptom Changes*). According to participants' responses, these changes seem to be influenced by detachment, defusion, and acceptance process, which primarily indicate increased psychological flexibility. These results are in line with a handful of randomized controlled trials (RCTs) exhibiting the effectiveness of MBIs for in- and outpatients with SSDs on a broad range of symptom representations, including positive and negative symptoms, depression, anxiety, and disturbances in cognition (56, 57, 77, 110). Moreover, multiple trials have demonstrated increased scores for social functioning and quality of life after MBIs (56). Possible reasons might be that MBIs can successfully be applied in group settings, are less time-consuming since treatment effects have been detected already after 6-10 sessions, giving solid ground for the in-patient implementation (74–76), and also participants show higher commitment when compared to CBT-based approaches.

Furthermore, the present outcomes build on previous research (58, 59, 61) by contradicting the widespread assumption and myth that MBIs could lead to the deterioration of psychotic symptoms. For the current study, none of the 27 in-patients experienced a deterioration of symptoms or showed any adverse effects. Thus, careful adaptions of MBIs concerning the targeted population, assured by psychometric pre-tests, proved to be explicitly fruitful and beneficial (112), demonstrating MBIs safety and applicability in the implementation for in-patients with SSDs. In a next step, future research should aim to conduct RCTs assessing its effectiveness to active and passive control conditions and broaden the current understanding of underlying process variables that enhance the treatment outcome.

### Perception of Context and Transfer of Therapy to Everyday Life

The main findings within the functional domain were associated with the *perception of context* and *transfer to everyday life,* which are described as characteristics of the framework concept of the MBGT. For the *perception of context* category, participants' responses seem to be in line with previous research stating that explicit adaptations in the implementation process for SSDs are required in order to facilitate the *core elements* and *effects* of MBIs. Participants deliberately reported that constant verbal instructions, as well as the sound of the singing bowl before and after each exercise, gave room for the internal inquiry process. Both *context* variables endorsed mindful awareness by giving anchor stimulus to direct attention to the present moment. Besides surrounding sounds and the therapist, participants named the short session, as well as the group inquiry process at the end of each exercise as fruitful and beneficial, as stated in subcategory 3. These factors have also been reported in current literature, where the utilization of short duration guided mediations (< 15 min), avoidance of prolonged periods of silence, and continuous instructions in basic anchoring techniques, small group sizes, and simplification of exercises have been primarily recommended (62, 102) to mitigate the risk of symptom deterioration. Furthermore, a simple, not too abstract metaphoric language seems to be useful to avoid misunderstanding, (113) while enabling enough room after each exercise within the inquiry process. Conducting sessions in a group format provided an efficient way to provide MBIs to a higher number of in-patients while parallelly promoting room for social cohesion, inclusion, participation, and exchange of experiences. Recent research already demonstrated that mindfulness could increase empathy, social integration, and contact, while reducing loneliness (114, 115), altogether building the foundation of resonance with the environment. Through the assessment of qualitative interviews, participants described that the group format supported mindful activities, such as taking a mindful walk in a park nearby or mindful eating together, which in turn helped to overcome personal and social barriers. MBIs seem to be explicitly promising in the treatment of negative symptoms such as blunted affect, alogia, anhedonia, or asociality. However, future research is needed to examine the association between *perception of context* factors, the implementation, and the underlying efficacy of mindfulness.

As the last domain, the *transfer to the everyday life* of mindfulness has been named by the participants. Generally, most participants reported the wish to be able to transfer the exercises into their everyday life. During their in-patient stay, several patients started to cultivate mindfulness into their daily routine by taking mindful walks or eating mindfully with other in-patients as depicted in subcategory 3. These steps enabled processes of self-efficacy and empowerment, which is highlighted in subcategory 4, indicating that even short daily exercises, such as counting the breath or taking showers mindfully resulted in symptom decline. In this category, other participants emphasized the utility, simplicity, and practicability of MBIs, especially in comparison to other interventions such as Progressive Muscle Relaxation (PMR). Interestingly, only a minor number of participants had heard about mindfulness previously; however, no one had any personal experiences beforehand, indicating that a potentially valuable and effective intervention had not been implemented in the treatment of SSDs. The reasons may lay in the persistent myth that mindfulness might lead to an exacerbation of symptoms, even though the present study outcomes, as well as the current literature, demonstrate that MBIs are safe, applicable, well-tolerated, and lead to subjective symptoms reduction and increased well-being even after a few sessions conducted (54, 55, 74).

### Limitations

Whereas the present study aimed to explore a relatively unnoticed research field, some limitations should be taken into account. As patients with SSDs can have a high level of mistrust and paranoia as symptoms of the disorder, the use of digital recording media was omitted. Accordingly, only verbatim transcripts were made during the interviews. Furthermore, although initial evidence exists for the applicability and feasibility of MBIs in patients with severe positive symptoms, this study aimed to investigate their applicability in the context of in-patients who displayed residual symptoms with chronic symptom representation and primarily pronounced negative- and moderate psychotic symptoms. Also, due to the nature of this study, applicability was assessed in a limited setting. Therefore, to test for broader applicability, future studies should aim to investigate the potential applicability, feasibility, and acceptability in RCTs in different hospital settings. As this study did not aim to investigate long term effects of MBIs for SSDs, RCTs with pre-, post-, and follow-up measurements should be carried out. Moreover, future research will need control conditions to clearly differentiate outcomes. Based on the current design, it might be that personal accounts of participants before the intervention could have influenced the present results, which cannot be solely attributed to MBI. Therefore, upcoming trials should systematically examine the indication, effectiveness as well as possible adverse- and side effects of mindfulness for this population. Lastly, other factors influencing meaningful communication during the interviews might include florid psychotic symptoms leading to poor insight and communication. Furthermore, the side-effects of antipsychotic medication such as drowsiness cannot be ruled out. To address these issues, future trials should carefully monitor the medication regime and include less acute patients from outpatient facilities to allow generalizability of the results.

## Conclusion

The current study represents the first research on participant responses to MBI for in-patients with SSDs in a German psychiatric hospital setting. In order to capture the novelty of such an intervention, a qualitative approach was chosen, which allowed for in-depth exploration of participant experiences, perceptions, and responses, allowing for the exploration of possible mechanisms and processes. Particular chosen adaptations could confirm the initial applicability of the intervention for in-patients with SSDs. Based on the analysis of the in-patients' experiences, there is evidence that in-patients with SSDs display responses to the interventions which are embedded in the theoretical models of mindfulness. Importantly, participants stated that they experienced no signs of exacerbation or decompensation of psychotic symptoms after MBIs. Overall, participants reported positive emotional and metacognitive effects, changes in symptoms, and learned how to transfer these into their daily life practice.

## Data Availability Statement

The datasets generated for this study are available on request to the corresponding author.

## Ethics Statement 

The studies involving human participants were reviewed and approved by Ethikkommission der Charité - Universitätsmedizin Berlin. The patients/participants provided their written informed consent to participate in this study.

## Author Contributions

KB collaborated with the design of the study, conducted the interviews, and wrote the manuscript. AK conducted the interviews and the data analyses, wrote the manuscript. LF conducted the data analyses and edited the manuscript. FE conducted the interviews and edited the manuscript. NT, TT, and MB edited the manuscript. EH collaborated with the design of the study, the writing and editing of the final manuscript.

## Conflict of Interest

The authors declare that the research was conducted in the absence of any commercial or financial relationships that could be construed as a potential conflict of interest.
